# Effectiveness of innovative instructional module for professional competence in health literacy in medical students

**DOI:** 10.1186/s12909-022-03252-7

**Published:** 2022-03-28

**Authors:** Hui-Fang Yang, Chia-Chen Chang, Pei-Ling Tseng, Hsiang-Ru Lai, Jaw-Shiun Tasi, Wei-Hsin Huang, Yu-Hao Fan, Ci-Xiang Weng, Chen-Yin Tung

**Affiliations:** 1 Department of Health Promotion and Health Education, College of Education, National Taiwan Normal University, 162, Section 1, Heping E. Rd, Taipei City, 106 Taiwan; 2 Department of Family and Community Medicine, Tri-Service General Hospital, National Defense Medical Center, Taipei, Taiwan; 3 Department of Senior Citizen Service Business, College of Human Ecology and Design, St. John’s University, New Taipei, Taiwan; 4 Department of Family Medicine, National Taiwan University Hospital, Taipei, Taiwan; 5 Community Health Center, MacKay Memorial Hospital, Taipei, Taiwan; 6 Department of General Medicine, MacKay Memorial Hospital, Taipei, Taiwan

**Keywords:** Health literacy, Professional competence, Medical education, Medical student

## Abstract

**Background:**

Physicians should be equipped with professional competence in health literacy to communicate more effectively with patients with limited health literacy. However, the health literacy curriculum has not yet been refined globally, and is scarce in Taiwan’s medical education. We implemented an innovative instructional module to attain professional competence in health literacy among medical students and investigated its effects.

**Methods:**

We adopted a quasi-experimental design and recruited 204 fifth-year Taiwanese medical students between December 2019 and May 2020. Participants who worked as clerks at the Department of Family Medicine of three medical schools in northern Taiwan were assigned to the experimental group through convenience sampling. A total of 98 students received a three-hour innovative instruction, including medical simulation videos, role-playing, and board games. Both the experimental and control groups completed the online pre-test and mail-in post-test. A generalized estimating equation was applied to measure the effects of the intervention.

**Results:**

There was a significant difference between the experimental and control groups in terms of professional competence in health literacy in all three aspects. In terms of knowledge, the experimental group improved 12% more than the control group (𝛽=0.12, 95% CI: 0.05 ~ 0.19, *p* = 0.001). In terms of attitude, the experimental group improved by an average of 0.27 more points per question than the control group (𝛽=0.27, 95% CI: 0.08 ~ 0.46, *p* = 0.007). As for skill, the experimental group improved by an average of 0.35 more points per question than the control group (𝛽=0.35, 95% CI: 0.14 ~ 0.55, *p* = 0.001).

**Conclusion:**

The proposed innovative instructional module significantly improved fifth-year medical students’ professional competence in health literacy, which is expected to benefit their future medical practices.

## Background

Health literacy is pivotal for people to engage their knowledge, motivation and competencies to access, understand, appraise and apply health-related information to healthcare, disease prevention, and health promotion [1]. Many studies have suggested a general lack of health literacy among people across different countries. The WHO Regional Office for Europe conducted a large-scale adult health literacy survey and discovered that 29–62% of adults had insufficient or problematic health literacy [2]. 51.6% of adults in Taiwan have inadequate or problematic health literacy [3]. Studies have established a strong association between low health literacy and poor health outcomes, such as increased hospitalisation and utilisation of emergency care, poor use of preventative health services, inappropriate medication use, inadequate comprehension of medical information, decreased self-care ability, higher risk of contracting chronic diseases and poor prognosis, and unstable physical and psychological conditions [4–7].

Physicians in medical environments should be equipped with the ability to acquire better understanding, strengthen autonomy, and support the self-management of patients, which will in turn enhance their health literacy [8–11]. However, the gap between the health literacy evaluation of health care professionals and the actual health literacy skills of patients might cause difficulty in communication [12]. Health literacy practices play a key role in effective communication and lead to high-quality patient-centred healthcare [13, 14]. A crucial concern to address is the way future physicians can play a better role in providing health literacy. In addition, some barriers to health literacy practice in healthcare workplaces are resultant of lack of knowledge or training about health literacy and related activities [15]. School-based literacy educational interventions can be considered powerful tools to create positive impacts [16]. Therefore, health literacy training is imperative and should be incorporated into the medical student curricula.

Research projects in Taiwan and across the globe all suggest that the health literacy curriculum in current medical education has not yet been refined. Coleman et al. investigated 61 medical schools in the U.S. and found that 70% of them had introduced health literacy into their medical education curriculum, with content emphasis on “oral transmission of knowledge in health literacy” and “association among literacy, health literacy, and patient health status.” [17] Several reviews have reported that professional competence building in health literacy for undergraduate healthcare students and professionals could improve knowledge and skills and equip them to communicate effectively with patients [18–21]. Previous studies have shown the effectiveness of interventions on healthcare professionals’ professional competence in health literacy [22–25]. However, the lack of a comparative group was noted in previous intervention studies, which revealed insufficient evidence to support the effectiveness of the training curriculum. In the recent years, some research on training in health literacy focused on medical students and revealed a positive effect, but lacked comprehensive intervention approaches or evaluation methods [26–29].

There is a dire need to develop innovative teaching methods and empowerment dedicated to physicians’ professional competence in health literacy as awareness about health literacy issues and courses to mitigate the same are scarce in Taiwan’s medical education system. We systematically and rigorously established indicators of professional competence in health literacy, designed a validly structured questionnaire, and developed a theory-based, innovative instructional module to teach medical students how to observe and assess a patient’s health literacy, understand its significance, and provide suitable doctor-patient interaction patterns to patients with different levels of health literacy [30, 31].

Our goal was to implement an innovative instructional module in the medical school curriculum to teach medical students and investigate whether the intervention helped them improve their professional competence in health literacy.

## Methods

### Subjects and methods of study

**Fig. 1:**
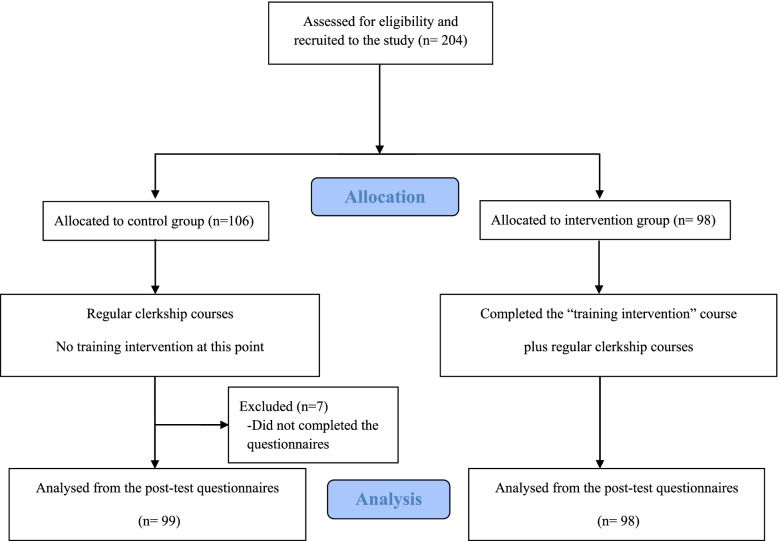
Flowchart of students throughout the study

We adopted a quasi-experimental design and recruited fifth-year students from 12 medical schools in Taiwan. The inclusion criteria were that the participants were fifth-year medical students willing to participate in the study. The exclusion criterion was that the participants did not complete the questionnaires. The sample size was determined using G-power analysis and based on a previous recent similar study [32]. Given a level of significance of alpha = 0.05, statistical power level of 0.8, and a medium effect size of 0.25, the minimum required sample size was 98, with 49 in each group being sufficient for a significant analysis. The requirement was 196 students in order to account for a dropout rate of 50% in the follow-up measurements. A flowchart of participant recruitment and allocation is presented in Fig. 1.

We employed convenience sampling and assigned 98 fifth-year medical students serving clerkships at the Department of Family Medicine in three teaching hospitals in northern Taiwan as the experimental group. All three hospitals were medical centres and referral hospitals, including two governmental and one private hospital. Participants attended a three-hour course on professional competence in health literacy, and completed a pre-test questionnaire prior to the course and a post-test questionnaire after it. A total of 106 fifth-year medical students from other medical schools were assigned as the control group through social media. They completed a pre-test via an online questionnaire and post-test via an email questionnaire 2 weeks later, and received no treatment regarding health literacy. All participants were informed about the research goal and execution method and provided signed consent. The teaching interventions and data collection were conducted from December 2019 to May 2020. All 98 participants in the experimental group completed the “Innovative Instructional Module for Professional Competence in Health Literacy” course as well as both pre-test and post-test. Of the 106 participants in the control group, 99 completed the pre-test and post-test. Valid response rates were 100 and 93.4%, respectively.

### Research instruments

We developed our questionnaire based on the indicators of physicians’ professional competence in health literacy, as proposed by Liu et al. [30] We referred to Coleman et al. [33] for health literacy teaching and practices and examined the Objective Structured Clinical Examination (OSCE) curriculum implemented across Taiwanese medical schools for clinical teaching [34]. Medical simulations of doctor-patient interactions for pre-test and post-test evaluation were subsequently designed. The questionnaire comprised of 47 items, including seven knowledge items, eight attitude items, and 32 skill items. The knowledge items were multiple-choice questions with a singular correct answer; a higher correct response rate (range 0–1) indicates a higher level of understanding of health literacy. Both attitude and skill items were on of 5-point Likert scale. Attitude items offered the answer options of “strongly disagree,” “disagree,” “no comment,” “agree,” and “strongly agree,” and scored between 1 and 5 points respectively (range 1–5 for each question), while skill items were answered in “very unconfident,” “unconfident,” “somewhat confident,” “confident,” and “very confident,” and scored between 1 and 5 points respectively (range 1–5 for each question). Higher scores in attitude questions indicate more agreement with the acceptance of health literacy concepts and respect for patient-centred health care. Higher scores on the skill questions indicated greater skill confidence in communication and interaction with patients.

We also submitted our questionnaire for content validity evaluation using the Content Validity Index (CVI). We invited six field experts to review the questionnaire content and evaluate the items and simulations in terms of their adequacy, significance, and clarity. For each item, the CVI was calculated by dividing the number of experts who gave a rating of 3 or higher by the total number of experts. The average CVI value was 0.8 or higher, with a Cronbach’s α value of 0.944, which demonstrated that our proposed questionnaire is an evaluation measure of good content validity.

### The instructional module, teaching curriculum, and instructor

Our proposed innovative instructional module and intervention strategies were developed based on the Spreitzer’s Psychological Empowerment Scale [31]. The intervention course module included two parts – two 80-min instructional sessions and two 10-min sessions for the pre-test and post-test surveys – for a total of 180 min. Teaching strategies involved didactic teaching, observational learning, inquiry-based learning, role-play, and game-based learning. We also created simulation videos using original content and invited practising physicians to demonstrate appropriate and inappropriate doctor-patient interactions to inspire student reflection on health literacy competence. Additionally, we developed a set of card-based board games, wherein students enacted the roles of physicians, patients, and observers. Students practiced teach-back and made shared decisions by performing in groups. The highlights of the teaching content are listed in Table 1. The teaching instructors were three senior attending physicians in family medicine from three different hospitals who were qualified to teach at universities. They all participated in curriculum development and teaching design, and performed pilot teaching and attended evaluation meetings, which created consistency between the teaching module’s curriculum and teaching execution.

**Table 1:** Overview of the Intervention Program

Session	Learning objectives	Contents	Teaching methods
1.Physician’s professional competence in health literacy (80 min)	-Concepts of physician’s professional competence in health literacy -Importance and value of physician’s professional competence in health literacy -Awareness and evaluation of patient’s health literacy -Empathy and acceptance -Respect and support	1. Present charts and figures on the problem with insufficient health literacy; illustrate common signs of insufficient health literacy in patients. 2. Use slide decks to introduce evaluation tools for health literacy that are used in Taiwan and other countries.	-Didactics -Observation learning -Group discussion -Problem solving - Inquiry-based learning
		1. Show videos that help students understand the sense of insecurity or embarrassment that patients may experience when receiving medical attention; encourage students to acknowledge a patient’s emotional reactions and express empathy so the patient feels supported and on the same front as their healthcare provider; evaluate a patient’s health literacy and accordingly adopt an adequate interaction pattern and offer social support. 2. Utilize videos with storylines centered around a crisis conceptual model on health literacy to help students see different medical scenarios that result from different levels of a physician’s professional competence on health literacy. The purpose is for students to acknowledge the importance of a physician’s ability to observe and assess a patient’s health literacy.	
		1. Present slide decks to summarize and conclude key points to help students acknowledge the importance of a physician’s ability to observe and assess a patient’s health literacy.	
2.New guideline for doctor-patient communication (80 min)	-Empathy and acceptance -Respect and support -Communication environment -Relationship building -Verbal and non-verbal communication -Easy-to-understand patient education materials and human resources -Teach-back -Medical purpose and needs -Shared decision-making -Confirm medical decision	1. Divide students into groups for quiz competition and role play, in which students will choose cards corresponding to the story plot; the purpose is to emphasize how different communication patterns will lead to different doctor-patient interaction and help students acquire adequate communication skills. 2. Present slide decks to conclude that good doctor-patient communication is the most important step towards a good doctor-patient relationship; practice applying “teach back” and “shared decision-making” to help patients obtain adequate medical services and the best healthcare outcomes.	-Didactics -Observation learning -Group discussion -Game-based learning -Role play
		1. Have students act out exemplary doctor-patient interaction scenes; the student who plays the observer role follow the checklist items to conduct evaluation and offer feedback; topics include “teach back,” “shared decision-making,” and “communication and interaction.	
		1. Use slide decks to summarize learning objectives for physician’s professional competence in health literacy; students should be able to apply relating principles in future medical scenarios to improve doctor-patient relationship and healthcare quality.	

### Data analysis

After collecting and filing the pre- and post-test questionnaires, we used SPSS version 22 (IBM SPSS Statistics for Windows, Armonk, NY, USA) for statistical analysis, utilising mean values and standard deviation to describe the distribution of variables and basic information. Knowledge items are expressed in terms of the average correct response rate while attitude and skill items are expressed in terms of the average score per item. In our statistical analysis, we performed an analysis of covariance with pre-test score as the covariate and post-test score as the dependent variable to test for within-group homogeneity of the regression coefficient. We found significant effect in “group*pre-test” interaction, suggesting that the two groups are not homogeneous. Subsequently, GEE was applied to compare the treatment effects between the different groups.

## Results

A total of 197 medical school students participated in this study, with 98 in the experimental group and 99 in the control group. Among them, 136 (69.0%) were male and 61 (31.0%) were female; in terms of educational background, 159 (80.7%) were enrolled in public schools and 38 (19.3%) in private schools. The study did not find a statistically significant difference in the distribution of sex or educational background between the two groups.

**Fig. 2:**
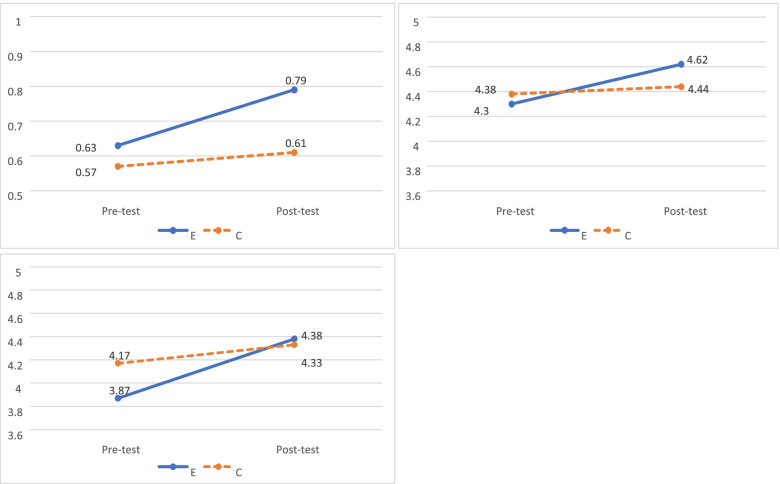
**A** Change in the mean proportion of correct answers for knowledge aspect in health literacy competence between the two groups. **B** Change in the mean scores of attitude aspect in health literacy competence between the two groups. **C** Change in the mean scores of skill aspect in health literacy competence between the two groups. E: Experimental group, C: Control group

Tables 2 and 3 illustrate the descriptive statistics and analysis results of the two groups’ pre-tests and post-tests. In terms of knowledge, the average correct response rate for the experimental group was 63% (SD = 18%) in the pre-test, 79% (SD = 20%) in the post-test; for the control group, it was 57% (SD = 16%) in the pre-test and 61% (SD = 19%) in the post-test. We applied GEE to analyse variables that impact the effect of intervention; after controlling for “group” and “test type” variables, the progress margin for the experimental group was 12% higher than the control group, with significant difference (*p* = 0.001). This suggests that the treatment significantly elevated the professional competence of the research subjects in health literacy (Fig. 2A).

**Table 2:** Mean proportion of correct answers and scores of professional competence in health literacy for post-test and pre-test of two groups

	Experimental group		Control group	
	Pre-test	Post-test	Pre-test	Post-test
	M (SD)	M (SD)	M (SD)	M (SD)
**Knowledge (range 0–1)**	0.63 (0.18)	0.79 (0.20)	0.57 (0.16)	0.61 (0.19)
**Attitude (range 1–5)**	4.30 (0.47)	4.62 (0.42)	4.38 (0.49)	4.44 (0.56)
**Skill (range 1–5)**	3.87 (0.43)	4.38 (0.49)	4.17 (0.54)	4.33 (0.61)

*M* Mean, *SD* Standard deviation

**Table 3:** GEE Analyses with comparisons of post-test and pre-test between two groups

	𝛽	95% CI	***p*** value
**Knowledge**			
Group (EG vs. CG)	0.06	0.01 ~ 0.10	0.018
Time (Post-test vs. Pre-test)	0.04	−0.01 ~ 0.09	0.134
Group^b^Time ^a^	0.12	0.05 ~ 0.19	**0.001**
**Attitude**			
Group (EG vs. CG)	−0.09	−0.22 ~ 0.05	0.207
Time (Post-test vs. Pre-test)	0.06	−0.09 ~ 0.20	0.437
Group^b^Time ^a^	0.27	0.08 ~ 0.46	**0.007**
**Skill**			
Group (EG vs. CG)	−0.31	−0.44 ~ − 0.17	< 0.001
Time (Post-test vs. Pre-test)	0.16	0.001 ~ 0.32	0.049
Group^b^Time ^a^	0.35	0.14 ~ 0.55	**0.001**

*EG* Experimental group, *CG* Control group

^a^Reference is “CG x Pre-test”. Interaction between Group

^b^Time means compare the degree of change between two groups

In attitude questions, the average score of the experimental group was 4.30 (SD = 0.47) in the pre-test and 4.62 (SD = 0.42) in the post-test; for the control group, it was 4.38 (SD = 0.49) in the pre-test and 4.44 (SD = 0.56) in the post-test. We applied GEE to analyse variables that impact effect of intervention; after controlling for “group” and “test type” variables, and the progress margin for the experimental group was 0.27 points higher than the control group, with significant difference (*p* = 0.007). This suggests that the treatment significantly elevated the professional competence of research subjects in health literacy (Fig. 2B).

In skill questions, the average score for the experimental group was 3.87 (SD = 0.43) in the pre-test and 4.38 (SD = 0.49) in the post-test; for the control group, it was 4.17 (SD = 0.54) in the pre-test and 4.33 (SD = 0.61) in the post-test. We applied GEE to analyse variables that impact the effect of intervention; after controlling for “group” and “test type” variables, the progress margin for the experimental group was 0.35 points higher than the control group, with significant difference (*p* = 0.001). This also suggests that the treatment significantly elevated the professional competence of research subjects in health literacy (Fig. 2C).

## Discussion

This study revealed that medical students in the experimental group showed a significantly increased in professional competence in health literacy for knowledge, attitude and skill compared to the control group after receiving teaching intervention. To our knowledge, this study is the first research project in Taiwan to target medical students in an intervention study on professional competence in health literacy that applies scale-based evaluation instrument, conducts teaching interventions, and achieves significant results.

There are several possible reasons for this effect. First, the proposed curriculum adopted multidimensional indicators, such as conception and evaluation, empathy and acceptance, communication and interaction, as well as medical information and shared decision-making. Other previous studies did not include such comprehensive indicators and most lacked shared decision-making aspects [26–29, 35, 36]. Second, we applied various student-centric teaching methods and created interesting materials for innovative teaching, including inquiry-based learning, observational learning, group discussion, game-based learning, and role-play. Strategies such as inquiry-based learning and role-play inspire students to think critically about a physician’s professional competence in health literacy. Original simulation videos and intriguing board games piqued their interest and motivation in this field. Third, we employed game-based learning in our doctor-patient interaction curriculum to highlight how different communication patterns lead to varied doctor-patient interactions. Meanwhile, the teach-back approach checks whether patients have fully comprehended a medical diagnosis or report. Fourth, according to Nutbeam’s model, there are three levels of health literacy – functional, interactive, and critical health literacy [37]. For healthcare professionals, physicians should consider a patient’s reading and writing abilities and whether they can exercise functional health literacy in regular doctor-patient interactions. In the course, the program teaches students about available measurements to assess a patient’s health literacy and common signs of insufficient health literacy. Interactive health literacy helps physicians and patients extract information from different communication channels, understand its significance, apply the information, and improve doctor-patient communication patterns. Our educational videos were physician centric. They presented outcomes in two-sided arguments to teach students about the consequences of having (positive) and neglecting (negative) professional competence in health literacy. This reinforces the students’ concerns and impressions regarding the issue. We also introduced the concept of shared decision-making for the best healthcare outcomes [38]. As for the highest level, critical health literacy, physicians provide patient-tailored teach-back approaches, provide readable materials and social support resources, and help patients execute self-management and disease management [39]. Our findings add to previous review articles and evidence that reveal the effectiveness of teaching module interventions in professional competency among medical students [18–21]. Other studies on health literacy training in medical students have shown positive effects, even in community-based service learning, but all of them were one-group pretest-posttest designs [35, 36]. Similar concepts of Nutbeam’s model were also applied in a study with a randomized controlled trial design, different from our quasi-experimental design [32]. This also emphasised that three levels of health literacy (functional, interactive, and critical) were embedded in the teaching module, so it included the shared decision-making aspect. The primary outcome variables evaluated were knowledge, attitude and skill competency using a valid questionnaire. However, our questionnaire corresponding to health literacy competency was designed with four clinical scenarios of physician-patient communication, and it woud be better to understand of the core meanings for each evaluation item instead of containing only straightforward questions.

Since the COVID-19 pandemic outbreak in early 2020, healthcare professionals and the public have been receiving disease information that caused swift and fundamental influence regardless of their accuracy. In a time like this, health literacy has become more important than ever [40, 41]. Misinformation threats have impacted all aspects of our social-ecological model, and the complexities of healthcare systems are increasing [42]. The need to establish health literacy systems of care and increase organizations’ health literacy sensitivity is dire [15, 43]. This study emphasises that the importance of teaching and intervention effects of health literacy courses in professional healthcare education is a key step in gradually reducing barriers to organizational health literacy practice [15].

.As for research limitations, first of all, since our control group recruited free-willed participants, it is possible that they were students interested in the subject matter, which could have given them better pre-test scores in attitude and skills in comparison to the intervention group. Second, a selection bias might have existed because of convenience sampling. Third, post-test surveys were completed immediately after the teaching intervention, therefore, long-term effects such as medical students’ behaviour change in the future medical residents or patients’ health outcomes have yet to be observed. Fourth, since our evaluation survey was self-administered, it is possible for self-reported errors or overestimation because participants perceived their capacity to be elevated over the time or from a learning effect to impact performance analysis. Fifth, our findings may not be generalizable to other regions of medical schools because we studied in northern Taiwan.

Based on our findings, regarding the curriculum reform of liberal arts courses, for medical students, we noted that although our current medical education offers extensive liberal arts courses, it falls short on issues concerning physicians’ professional competence in health literacy. We recommend transforming our proposed innovative instructional module into themed micro-teaching activities or dividing them into different sessions to streamline class time. We plan to conduct relevant courses in Taiwanese medical schools. We suggest hosting educational training in health literacy to raise physician awareness of the phenomenon of low health literacy in Taiwanese clinical fields. Government departments and academic institutes can also develop teaching materials that physicians across specialties may take advantage of during healthcare services or health education scenarios to improve and raise awareness of patients’ health literacy. They will provide access to appropriate medical information to enhance doctor-patient professionals and improve the quality of care.

## Conclusions

The proposed innovative instructional module significantly improved fifth-year medical students’ professional competence in health literacy, which is expected to benefit their future medical practice. They should be able to observe a patient’s health literacy more acutely to help them obtain, understand, evaluate, and apply medical information; achieve efficient utilisation of medical resources; and improve healthcare quality.

## Data Availability

The datasets generated during and/or analyzed during the current study available from the corresponding author on reasonable request.

## Abbreviations

COVID-19: Coronavirus disease 2019
OSCE: Objective Structured Clinical Examination
CVI: Content validity index
GEE: Generalized estimating eq.
SD: Standard deviation
